# Do iPads promote symbolic understanding and word learning in children with autism?

**DOI:** 10.3389/fpsyg.2015.00138

**Published:** 2015-02-12

**Authors:** Melissa L. Allen, Calum Hartley, Kate Cain

**Affiliations:** Department of Psychology, Lancaster University, LancasterUK

**Keywords:** iPad, autism spectrum disorders (ASDs), word learning, symbolic understanding, picture-based learning

## Abstract

The use of the Apple iPad has skyrocketed in educational settings, along with largely unsubstantiated claims of its efficacy for learning and communication in children with autism spectrum disorder (ASD). Here, we examine whether children with ASD are better able to learn new word–referent relations using an iPad or a traditional picture book. We also examine the hypothesis that presenting multiple, differently colored, exemplars of a target referent will promote adaptive label generalization compared to the use of a single exemplar. Sixteen minimally verbal children with ASD were taught a new word in four within-subjects conditions, which varied by media (iPad vs. book) and content (single vs. multiple exemplar presentation). Children were then tested on the ability to symbolically relate the word to a 3-D referent (real-life depicted object) and generalize it to a differently colored category member (another similarly shaped object). The extent of symbolic understanding did not differ between the two media, and levels of generalization did not differ across conditions. However, presentation of multiple exemplars increased the rate that children with ASD extended labels from pictures to depicted objects. Our findings are discussed in terms of the importance of content to picture-based learning and the potential benefits and challenges of using the Apple iPad as an educational resource for children with ASD.

## INTRODUCTION

Severe impairments in language acquisition and usage are a common characteristic of children diagnosed with autism spectrum disorder (ASD; Wing and Gould, 1979; Klin et al., 2002). Specifically, 80% of individuals with ASD aged 5-years and younger who enter special education are non-verbal (Bondy and Frost, 1994), and 30% of children with ASD are minimally verbal at 9-years (Anderson et al., 2007). The failure of these children to acquire spoken language has a devastating impact on their capacity to communicate, and picture-based methods such as the Picture Exchange Communication System (PECS; Bondy and Frost, 1994) have been widely implemented as an aid for expressive language. In general, pictures “are one of the most widely available and effective of all the teaching material genres” (Chang et al., 2005, p. 147), and provide a ubiquitous scaffold for learning about the world (Tare et al., 2010). Studies examining the efficacy of PECS have demonstrated that picture-based interventions can successfully facilitate communication in minimally verbal children with ASD (Flippin et al., 2010) particularly when trainers are guided on optimal delivery methods (Howlin et al., 2007).

The introduction of the Apple iPad in 2010 has seen a shift toward technology-mediated learning for typically and atypically developing children. Tablets and similar handheld devices offer the promise of flexible, mobile, and individualized learning to support language and literacy development, maths, social sciences, etc. (Banister, 2010) and there are an increasing number of software applications (“apps”) to support this. In 2012, there were ~1.5 million iPads being used in classrooms across America (Kessler, 2012), and the Apple Store now contains over 75,000 education apps (retrieved from http://www.apple.co.uk, 2014). However, to date, there is little empirical support that the technology, rather than the content, results in improved educational outcomes (Biancarosa and Griffiths, 2012), despite media reports to the contrary.

Our focus here is on the use of tablets (and apps) to foster language acquisition and communication in children with ASD. The effectiveness of apps as supports for language and communication in children with intellectual disabilities is implicitly assumed, as very few studies have investigated whether these children actually benefit from iPad-based learning relative to more conventional picture-based mediums (e.g., traditional picture books). In a recent review, Kagohara et al. (2013) described how iPods and iPads have been successfully used to teach communication skills to individuals with developmental disabilities, such as how to access preferred stimuli and request snacks. There are also reports that the iPad can successfully reduce challenging behaviors (Neely et al., 2013), and teach pretend play (Murdock et al., 2013), numeracy skills (Jowett et al., 2012), and picture naming (Kagohara et al., 2012). However, the extremely limited sample sizes of these studies (e.g., 1–4) limit the generalization of their findings, particularly when we consider the heterogeneity of individuals across the autism spectrum (Folstein and Rosen-Sheidley, 2001). Perhaps more critically, the extant literature has yet to address whether iPads can be used to effectively target core cognitive deficits in symbolic understanding and vocabulary acquisition.

On both theoretical and applied levels, it is vital to assess whether children with ASD (particularly minimally verbal individuals who rely on pictorial communication systems) understand symbols. If children are to become effective communicators and navigators of the social world, it is crucial that they successfully comprehend the varied symbol systems that pervade their environment (Happé, 1995). Alongside language, pictures are a class of symbols that play an extremely important role in children’s early learning. Pictures are visual representations of objects that exist independently in time and space, and they enable viewers to learn about reality without directly experiencing it.

From early childhood, children spend considerable time engaged in joint picture–book reading interactions with adults (Debaryshe, 1993). Within this context, adults name and provide facts about pictures with the good-faith expectation that children (a) understand that pictures represent real things, and (b) that they will generalize information to depicted objects in a manner consistent with mature symbolic understanding. In typical development, children realize that verbal labels paired with pictures normally refer to symbolized referents (rather than pictures themselves) by 18–24-months (Preissler and Carey, 2004; Ganea et al., 2009). By contrast, a growing collection of studies has identified fundamental differences in how children with ASD map and generalize word–picture relations.

Preissler (2008) demonstrated that children with ASD frequently map verbal labels onto individual pictures rather than symbolized referents, preventing generalization to real objects. However, this atypical behavior may be related to iconicity – the extent that a picture perceptually resembles its referent. In Preissler’s study, the pictorial stimuli were schematic black-and-white line drawings. Higher levels of perceptual similarity between picture and referent make the symbolic relationship more salient, increasing the likelihood that the viewer will map the correspondence between the two and draw inferences from one to the other (DeLoache, 1995). In their recent study, Hartley and Allen (2015) taught children with ASD a series of novel words paired with color and non-color pictures of unfamiliar objects. At test, children were asked to select the referent of the newly learned label from arrays consisting of the target picture paired with the depicted object and then a differently colored variant of the depicted object. The results revealed that children with ASD more frequently extend labels to objects depicted in color pictures than non-color pictures. However, they were still much less likely to do so than typically developing (TD) peers, suggesting that pictorial understanding is fragile in autism, and that variability in certain visual factors can promote or inhibit their comprehension (also see Hartley and Allen, 2014a,c).

In the current study we investigated whether the *medium* of presentation – traditional picture book vs. the iPad – influenced children’s acquisition of word–referent concepts. Books and iPads can both be read and interacted with alone or with others. For TD children, though, traditional picture books may facilitate learning (see Ganea et al., 2008) because they provide optimal opportunities for joint interaction and engagement, which are predictively related to language development (Bus et al., 1995; Blewitt et al., 2009). However, by definition, children with ASD are impaired in the domain of social-cognition (Klin et al., 2002) and may show an aversion to engaging in social-interactions (Sigman et al., 1986). For this reason, the potentially self-contained nature of the iPad might actually be a more comfortable environment and, therefore, a better source for learning (see Kagohara et al., 2013). In particular, the device enables the user to access both auditory and visual output and provides direct reinforcement for learning (e.g., flashes or sounds when a correct response is made). Of course, it is entirely possible that the self-contained aspect of the iPad technology may hinder spontaneous communication with others, which might be necessary to support the learning of novel symbol–referent relations. Recent work supports this viewpoint. A study of parent–child shared reading with TD 3- to 6-year-olds found that traditional print books and interactive e-books resulted in different patterns of engagement: the traditional print book resulted in more content-focused interactions between parent and child to support understanding, whilst the e-book resulted in more (physical) child–book interactions (Chiong et al., 2012). Thus, our first aim is to examine whether children with ASD learn words and symbolic picture–referent relations more successfully from an iPad or a traditional picture book.

A second factor that might affect symbolic understanding is the content of the teaching material itself. By around 24 months of age, TD children infer the general rule that noun-referent relations are constrained by shape, and thus generalize words to novel objects based on this feature, rather than color, size, or texture (Landau et al., 1988). In contrast, children with ASD do not show this ‘shape bias’ (Tek et al., 2008). This may be because they do not prioritize global shape (a category-defining detail) over category irrelevant details such as color (Hartley and Allen, 2014b) due to differences in visual processing (Frith and Happé, 1994) and categorization (Klinger and Dawson, 2001). However, previous research has shown that children with ASD perform better on word learning tasks when provided with differently colored exemplars during training (Klinger and Dawson, 1995; Nosofsky and Johansen, 2000). Presentation of multiple exemplars of word–picture mappings in which the target referent differs in color may serve to highlight similarity of shape, thus fostering shape-based generalizations. This hypothesis was tested in the current study.

This study addressed two research questions. First, we examined whether children with ASD are better at acquiring new vocabulary and conceptual knowledge of object categories from iPads or traditional picture books. Second, we evaluated whether presenting multiple, differently colored, pictures of a target object facilitated shape-based generalization. Critically, we focused on minimally verbal children with concomitant intellectual disabilities. This population was selected because (a) they are often neglected in empirical research despite representing a large proportion of the autism spectrum (Anderson et al., 2007), (b) children matching this profile are typical beneficiaries of picture-based educational practices and interventions, and (c) they are the target audience for the producers of picture-based communication apps on the iPad. Thus, the findings of this study will advance our theoretical understanding of autism and potentially reveal significant implications for clinical and educational practice.

Based on the paradigms of Preissler (2008) and Hartley and Allen (2015), children with ASD were taught a novel word paired with an iconic color photograph in four separate conditions: (1) via an iPad, repeatedly presenting a single representation of the target object, (2) via a picture book, repeatedly presenting a single exemplar, (3) via an iPad, presenting multiple differently colored representations of the target object, and (4) via a picture book, presenting multiple differently colored representations. This within-subject design allowed us to assess individual learning styles and to determine whether children show symbolic understanding across all conditions, or benefit from a specific medium/content combination. Audio stimuli were presented by the integrated microphone in iPad conditions, and by the experimenter in book conditions.

We predicted an advantage for learning from the iPad on the basis that its self-contained nature would provide a less stressful learning environment for children with ASD. More specifically, the reduced social-interaction demands associated with this medium may allow children to devote greater cognitive resources to learning. We also predicted an increase in shape-based label generalization when learning from multiple exemplars of a target referent, as this should highlight that shape defines referential word-picture-object concepts.

## MATERIALS AND METHODS

### PARTICIPANTS

Participants were 16 children with ASD (all male; *M* age = 9.6 years; SD = 4.0; range: 4.1–16.2 years) recruited from a specialist school in the North–West, UK. All children received an autism spectrum diagnosis from a qualified educational or clinical psychologist, using standardized instruments (i.e., ADOS and ADI-Revised; Lord et al., 2002; Rutter et al., 2003a). Diagnosis was confirmed via the Social Communication Questionnaire – Lifetime, completed by children’s parents (Rutter et al., 2003b; *M* = 27.5; SD = 5.9; range: 17–34). All children were recipients of picture-based communication interventions due to impaired expressive language skills (i.e., they were minimally verbal). In addition, all children were exposed to iPads within their educational setting for learning and reinforcement (e.g., preferred games). The mean receptive language age of the group was 3.9 years (SD = 1.4; range: 2–6.4 years)^1^, as measured by the British Picture Vocabulary Scale (Dunn et al., 1997). Children’s non-verbal intellectual abilities were measured using the Leiter-R (Roid and Miller, 1997), which revealed a mean IQ of 57.5 (SD = 19.6; range: 36–95), indicating concomitant intellectual disabilities (as is expected in a minimally verbal population).

### MATERIALS

**FIGURE 1:**
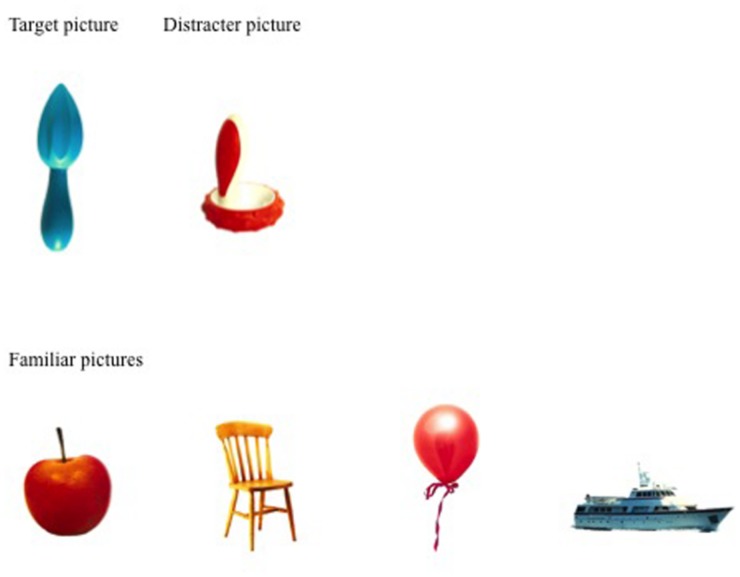
**Example stimuli set**.

Stimuli were color photographs presented via picture books and an Apple iPad2, and a variety of familiar and unfamiliar objects (see **Figure 1**). Four picture books were created – two “Single Exemplar” books (BookS1, BookS2) and two “Multiple Exemplar” books (BookM1, BookM2). Each book contained 13 training pictures and two test pictures. Training pictures were five photographs (sized 15 × 20 cm) of different familiar objects (see **Figure 1** for an example) and eight photographs of two unfamiliar objects (four pictures of each). In each book, one unfamiliar object was a ‘target object’ and the other was a ‘distracter object.’ Only one training picture was visible at a time. In the Single Exemplar books, the four training pictures of the target object were identical (e.g., a green rattle toy), as were the four pictures of the distracter object. In the Multiple Exemplar books, the four training pictures of the target object were differently colored (e.g., blue, purple, pink, and brown lemon juicers), but the four pictures of the distracter object were identical. Test pictures were slightly smaller (15 × 10 cm) photographs of the target and distracter objects that were positioned on opposite pages at the end of each book. The reduction in size was necessary to equate the Book and iPad conditions (iPad test pictures had to be smaller in order to fit them both on-screen simultaneously). In the Multiple Exemplar books, the test picture of the target object was the same color as one of the four training variants (selected at random). Additionally, a picture of each target object was printed and laminated for use at the test stages.

Four picture learning ‘lessons’ were created on an Apple iPad2 using See.Touch.Learn application software. These lessons mirrored the content and presentation of the four picture books described above – two were Single Exemplar lessons (iPadS1, iPadS2) and two were Multiple Exemplar lessons (iPadM1, iPadM2). Each lesson contained exactly the same pictures as one of the books described above (e.g., photographs of the same target object, distracter object, and familiar objects appeared in iPadS1 and BookS1). Training pictures on the iPad were presented individually and were sized 15 × 10 cm. Additionally, another picture of the target object (same dimensions as the training pictures) appeared after the test pictures for use at the subsequent stages. Audio stimuli were recorded using the iPad’s microphone.

Sixteen 3-D objects were used to assess learning. Four were the target objects depicted throughout Book/iPadS1 and Book/iPadS2, and in the test pictures of Book/iPadM1 and Book/iPadM2. Four were differently colored variants of each target object that were not depicted by any training pictures (‘novel objects’). Four were the depicted distracter objects, and four were familiar objects (model horse, toy telephone, baby book, cup) that were not depicted by any training pictures.

### PROCEDURE

Participants were tested individually over four sessions (~1 week apart) in their own schools. Children were seated at a table next to the experimenter, and were reinforced for attention and good behavior. Correct performance was only reinforced at the Word Learning Test (described below). The Leiter-R and BPVS were administered in separate individual sessions prior to testing.

In each session, children were taught a unique pairing between a photograph of an unfamiliar target object and a novel word (dax, wug, yat, ged). In two sessions, pictures of the target object were the same color throughout training (Single Exemplar trials; S1, S2), and in two sessions, children viewed four differently colored pictures of the target object (Multiple Exemplar trials; M1, M2). Children received one Single Exemplar trial in book form (BookS1 or BookS2) and the other in iPad form (iPadS1 or iPadS2). Similarly, children received one Multiple Exemplar trial in book form (BookM1 or BookM2) and the other in iPad form (IpadM1 or IpadM2). For example, a child might receive S1 and M2 in Book form (BookS1, BookM2) and S2 and M1 in iPad form (iPadS2, iPadM1). The order and form in which children received S1, S2, M1, and M2 were counterbalanced. Sessions consisted of a Training Stage, a Word Learning Test, a Mapping Test, a Perseveration Control, an Object Bias Control and a Generalization Test (experienced in a fixed order).

## TRAINING STAGE

### BOOK CONDITIONS

The picture book was placed in front of the child and the experimenter turned the pages. The experimenter pointed to every training picture, and also verbally directed the child’s attention. Pictures of the target object were labeled twice using a novel word (e.g., “Look, it’s a dax. See, the dax.”), pictures of familiar objects were labeled once (e.g., “Look, it’s a ball.”) and pictures of the distracter object were not labeled (e.g., “Look at this.”). Pictures of the same type were never presented consecutively (e.g., children never viewed the target object followed by another picture of the target object).

### iPad CONDITIONS

The iPad was placed in front of the child. When a training picture appeared, the child heard an audio recording of the experimenter. As in the Book conditions, pictures of target objects were labeled twice, pictures of familiar objects were labeled once, and pictures of distracter objects were not labeled. Unlike the Book conditions, children controlled their transition between pictures; after viewing a picture and hearing its label, the child pressed an on-screen button to bring up the next picture^2^. The experimenter sat quietly while the participant was engaged in the ‘lesson’ and offered verbal reinforcement for good attention when necessary.

### ASSESSMENTS OF LEARNING

Learning was assessed with five tests: the Word Learning Test, Mapping Test, Perseveration Control, Object Bias Control, and Generalization Test. These were first scored individually for each condition following Hartley and Allen (2015). In accordance with Preissler’s (2008) coding criteria, only intentional responses were coded (e.g., giving or sliding an item to the experimenter, pointing to or picking up and showing the experimenter an item).

### WORD LEARNING TEST

At the end of the Training Stage, children were presented with two adjacent pictures depicting the target and distracter objects, and were asked to identify the referent of the newly learned word (“show me the dax”). In both the book and iPad conditions, children responded by pointing to or touching their chosen picture respectively. Selection of the target object confirmed that children had mapped the correct word–referent relation, and was rewarded with positive reinforcement. This was scored as a ‘pass.’ If children selected the distracter object, they were given corrective feedback (“Actually, this is a dax [pointing to target object]. Let’s play the game again!”) and the Training Stage was repeated until they correctly selected the target object. The number of times it took children to successfully complete the Training Stage was noted.

### MAPPING TEST

Following a correct response on the Word Learning Test, children were presented with a picture of the target object and the real 3-D target object, and were asked to identify the referent of the newly learned word (“show me a dax”). In both the book and iPad conditions, the picture was presented as a flash card or on-screen respectively (this was the case for all subsequent tests). If a child learned the label associatively, without understanding the symbolic word-picture-object relations, they would be expected to select the picture alone. After making their selection, the experimenter removed the stimuli from the child’s line of sight and recorded the selection. Responses were coded as ‘target picture only,’ ‘target object only,’ or ‘both picture and object.’ Selecting the ‘target picture only’ was classed as an ‘associative’ response. Since people use the same label to describe both real and depicted versions of the same object, ‘target object alone’ and ‘both picture and object’ were considered ‘symbolic’ (see Ganea et al., 2009). The Perseveration Control followed immediately after the child’s response.

### PERSEVERATION CONTROL

Based on evidence that children with ASD often form associative word–referent mappings (e.g., Baron-Cohen et al., 1997; Preissler and Carey, 2005; Preissler, 2008), we expected that many participants would select the picture at the Mapping Test. However, selection of the picture could also be due to perseveration (e.g., Shu et al., 2001) elicited by positive reinforcement at the Word Learning Test. Thus, children were presented with a picture of the target object and a previously unseen 3-D familiar object, and were asked to identify the familiar item (“show me a horse”). Selection of the familiar object was scored as a ‘pass.’ Selection of the target picture indicates perseverative responding, rather than associative word learning. Selection of the familiar item shows that (a) children are capable of switching between responses, and (b) selection of the target picture at other test stages is due to associative word learning. The Object Bias Control followed immediately after the child’s response.

### OBJECT BIAS CONTROL

Children’s repeated selection of objects could be due to a preferential bias for 3-D items over pictures. That is, they may select the object stimulus in each trial simply because it is more attentionally salient than the picture, not because they understand symbolic word-picture-object relations. Children were presented with a picture of the target object and the 3-D distracter object, and were asked to identify the referent of the newly learned word (“show me a dax”). Selection of the target picture was scored as a ‘pass.’ Incorrect selection of the distracter object indicates an object-selection bias. The Generalization Test followed immediately after the child’s response.

### GENERALIZATION TEST

Children were presented with a picture of the target object and a differently colored variant of the depicted object (novel object), and were asked to identify the referent of the newly learned word (“show me a dax”). Understanding that pictures communicate information about categories rather than specific exemplars would direct children to extend the label to the novel object. Conversely, associative word–referent mapping or the belief that pictures represent specific exemplars (i.e., matching on shape and color) would prevent label extension to the novel object. Children’s responses were coded as ‘target picture only,’ ‘novel object only,’ or ‘both picture and object.’ Responses that included the novel object (‘novel object only’ or ‘both picture and object’) indicated successful generalization of the label to a differently colored category member. The test session ended after the child’s response.

## RESULTS

For ease of interpretation, the findings for each condition are presented within each different trial type.

### WORD LEARNING TEST

Every participant passed the Training Stage of each condition before proceeding to the test questions. Children selected the correct picture at the first opportunity on 55/64 (86%) of trials, and after two repetitions of the Training Stage on the remaining 9/64 trials (14%). A repeated measures analysis of variance (ANOVA) confirmed that there were no differences between the number of presentations required across conditions, *F*(3,45) = 0.135, *p* = 0.94, $\eta_{p}^{2}$ = 0.01.

### MAPPING TEST

**FIGURE 2:**
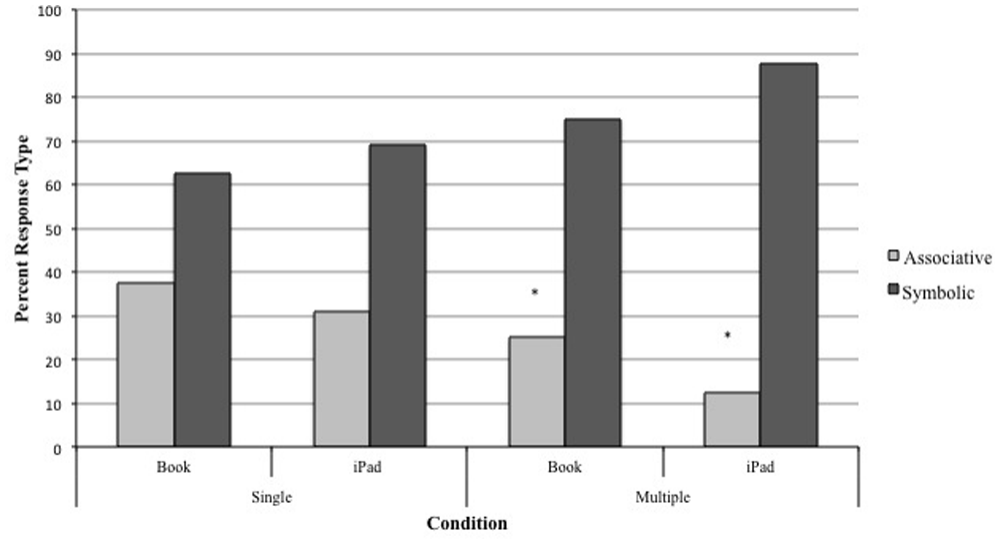
**Percentage of symbolic/associative responses in the Mapping Test across all conditions.** *p < 0.05.

The percentage of target picture only, target object only, and both responses for each condition are displayed in **Table 1**. There were no significant differences between these three response types across conditions. However, this does not tell us whether the children are learning associatively or symbolically because a selection of the object alone or both actually supports symbolic understanding. The next analyses therefore address the two learning styles (associative vs. symbolic) using a chance rate of 50%. When associative and symbolic responses were compared, children only made symbolic responses at above-chance rates in the Book-Multiple, χ^2^ (1, *N* = 16) = 4, *p* = 0.046, and iPad-Multiple conditions, χ^2^ (1, *N* = 16) = 9, *p* = 0.003, suggesting that their understanding of word-picture-object relations was facilitated by multiple exemplars (see **Figure 2**). When symbolic responses from the two Single conditions were summed and compared to these responses from the two Multiple conditions, a paired *t*-test revealed a borderline significant difference, *t*(15) = 2.1, *p* = 0.055, *d* = 0.42 (Multiple > Single). Responses from book and iPad trials did not differ. Thus, it appears that children with ASD are more likely to extend labels from pictures to referent objects when multiple exemplars are presented, regardless of the medium of presentation.

**Table 1:** Participants’ responses at the Mapping Test and Generalization Test in each condition.

Condition	Extension test	Target picture only (%)	Target object only (%)	Both picture and object (%)
Book-Single	Mapping Generalization	37.6 43.8	31.2 25	31.2 31.2
iPad-Single	Mapping Generalization	31.2 37.5	43.8 43.8	25 18.7
Book-Multiple	Mapping Generalization	25 43.8	50 18.7	25 37.5
iPad-Multiple	Mapping Generalization	12.5 43.8	56.3 25	31.2 31.2

### PERSEVERATION CONTROL

Children passed the perseveration control by selecting the familiar object and disengaging from the previously reinforced target picture on 63/64 trials (98.4%).

### OBJECT BIAS CONTROL

Children passed the object bias control on 81.3–87.5% of trials across conditions. These rates were all significantly above-chance (χ^2^ = 6.2–21.1, all *p*s < 0.01).

### GENERALIZATION TEST

The percentages of target picture, novel object, and both responses for each condition are shown in **Table 1**. There were no significant differences between these three response types across conditions using a Friedman’s test, the non-parametric equivalent of a repeated measures ANOVA, [χ^2^(3) = 1.3, *p* = 0.72]. Overall, children generalized labels from target pictures to differently colored category members (novel object only and both responses collapsed) on 9/16 (56.3%) trials in Book-Single, Book-Multiple, and iPad-Multiple conditions, and 10/16 (62.5%) in the iPad-Single condition. None of these rates significantly exceeded chance.

### RELATIONS BETWEEN SYMBOLIC RESPONDING AND PARTICIPANT CHARACTERISTICS

Correlation and regression analyses were conducted to determine whether chronological age, receptive language, non-verbal IQ, or autism severity were related to symbolic understanding of word-picture-object relations. When conditions were collapsed, there was a significant positive correlation between non-verbal abilities and label extension to depicted referents, *r*(14) = 0.65, *p* = 0.007. There was also a significant negative correlation between chronological age and label extension, *r*(14) = –0.52, *p* = 0.039, suggesting that younger children were more likely to respond symbolically. To establish which variable(s) significantly predicted symbolic responding, the above factors were entered into a stepwise regression. The regression yielded a significant model (*F* = 4.3, *p* = 0.038) containing only non-verbal IQ (β = 0.56), which accounted for 68% of variation in performance (*R*^2^ = 0.68).

Pearson’s correlations were also performed between the background measures and symbolic performance across the single and multiple conditions, as these were the trial types that elicited differences in symbolic responding; see **Table 2**. In line with the above analyses, non-verbal abilities were significantly related to symbolic performance across single and multiple trials, and chronological age was negatively related to performance in the multiple condition.

**Table 2:** Correlations between symbolic understanding in the Mapping Test of Single and Multiple trial types, and CA, MA, NVIQ, and autism severity (SCQ).

	Single	Multiple	CA	MA	NVIQ	SCQ
Single						
Multiple	*r* = 0.687**					
	*p* = 0.003					
CA	*r* = –0.449	*r* = –0.508*				
	*p* = 0.081	*p* = 0.044				
MA	*r* = 0.357	*r* = 0.343	*r* = 0.150			
	*p* = 0.175	*p* = 0.194	*p* = 0.578			
NVIQ	*r* = 0.666**	*r* = 0.516*	*r* = –0.398	*r* = 0.195		
	*p* = 0.005	*p* = 0.041	*p* = 0.127	*p* = 0.468		
SCQ	*r* = 0.152	*r* = –0.180	*r* = 0.105	*r* = –0.052	*r* = –0.428	
	*p* = 0.619	*p* = 0.557	*p* = 0.732	*p* = 0.865	*p* = 0.145	

CA, chronological age; MA, mental age determined by the BPVS; NVIQ, non-verbal intelligence quotient determined by the Leiter-R; autism severity determined by the Social Communication Questionnaire.

*p < 0.05, **p < 0.01.

## DISCUSSION

This study investigated (a) whether children with ASD are more successful at acquiring new vocabulary and object knowledge from iPads or picture books, and (b) whether the presentation of single or multiple referents influences adaptive label generalization. In a within-subjects design, participants were taught a series of novel word–picture pairings using single and multiple exemplars of depicted objects, presented via picture books and an iPad. Children’s learning, mapping, and generalization of word–picture relations were assessed. Importantly, the results of the mapping test revealed that medium of presentation – iPad or book – did *not* impact on children’s extension of labels from pictures to real objects. Rather, children with ASD only extended labels to depicted objects at above-chance rates in Multiple Exemplar trials, and tended to make fewer symbolic responses in Single Exemplar trials. Thus, the *content* being presented may be a more significant influence on children’s symbolic understanding of word-picture-object relations than the medium of presentation.

Contrary to our predictions we did not find an advantage for learning with the iPad. On the one hand, this is a reassuring finding: learning and symbolic understanding do not differ simply because of the *medium* of learning. Further, our finding is not actually at odds with media reports and other published research on interventions with iPads, because that other work has not compared learning between two different methods of presentation, rather it has demonstrated that children with ASD can learn from iPads (see Kagohara et al., 2013). On the other hand, there are good reasons to expect that the reduced social-interaction afforded by the iPad might enhance learning in children with ASD by reducing environmental stress and allowing cognitive resources to be dedicated to learning. Future research could usefully compare engagement and interaction during the learning phase to determine if this is influenced by the medium of delivery, which would provide greater insight into why and how it might impact on learning. Certainly, studies of TD children demonstrate differences in parent–child interactions between reading a book vs. an iPad, and these effects might be related to the increased story recall found for traditional story books (Chiong et al., 2012). Thus, investigating the social-interaction elements of learning, how they may differ between traditional books and iPads, and their effect on learning should be a focus for future research. One exciting way to analyze such differences is to use eye-tracking technology, which has been successfully used to determine children’s looking patterns within their picture communication systems (see Gillespie-Smith et al., 2014). This can inform understanding of both the process and outcome of learning.

Regardless of medium, we demonstrated that presenting multiple color photographs of target referents increases the likelihood that children with ASD extend newly acquired labels to referent objects. The advantage for multiple exemplars might have arisen because the process of pairing one verbal label with several differently colored pictures reduced the formation of associative word–picture relations by indicating that names can be extended to various items. By demonstrating that a single label does not refer to a unique referent (i.e., a specific target picture), the Multiple Exemplar conditions may have increased children’s awareness that words can be generalized to additional items in one’s environment, including perceptually similar objects. By contrast, in Single Exemplar trials, the process of repeatedly pairing a verbal label with one target picture may have narrowed the referential relation to the extent that the picture itself (rather than the depicted object) was more frequently considered the referent of the word (Plaisted, 2001; Hartley and Allen, 2015).

When learning new vocabulary, children with ASD often require significantly more word–referent pairings than TD children (e.g., Hartley and Allen, 2015), which naturally increases the risk of associative learning and inhibits generalization to novel referents (a common deficit in ASD). Thus, our finding that the presentation of multiple exemplars can promote label extension in contexts that potentially foster associative learning has important applied benefits. Specifically, if this strategy were to be integrated into clinical and educational practices (e.g., the delivery of PECS; the development of iPad communication apps), minimally verbal children with ASD may better understand that 2-D representations can refer to 3-D objects, leading to improvements in their ability to communicate using pictorial aids. The generation of multiple exemplars that differ simply by color is a feature that could easily be incorporated into educational software to support the learning of children with ASD. In this way, technology-mediated learning confers certain benefits over less flexible traditional hard copy learning materials because they allow adaptive features to suit an individual child’s progress.

Contrary to our predictions, teaching children with ASD new word–referent concepts using Multiple Exemplars did not foster shape-based generalization to novel category members. It is possible that children who extended labels to the target object at the Mapping Stage, but not the novel object at the Generalization Stage, may not understand that pictures serve a general referencing function (i.e., they can represent categories of objects, in addition to specific exemplars). They may also have difficulties processing similarities between visual stimuli (Plaisted, 2001). It will be important to promote categorical knowledge in children with ASD by flexibly pairing the pictures used in educational apps with multiple category members of real world objects to highlight the generality of pictorial symbols.

The correlations and regression analyses indicated a positive relationship between non-verbal IQ and symbolic understanding. One possibility is that those children with lower IQ may be more likely to learn words as one-to-one associative relations (preventing extension to other items), and less likely to understand their intended referential function. Relatedly, it may be that non-verbal IQ reflects the severity of children’s impairments in processes that underpin the formation of word–referent concepts, such as shape-based categorization and prototype formation (Younger, 1990; Klinger and Dawson, 2001). We also identified a negative relationship between chronological age and symbolic understanding. This may have emerged because the older children had more severely impaired non-verbal abilities (see **Table 2**), leading to increased associative learning. However, due to the small sample in the present study, we are unable to draw firm conclusions about causal mechanisms underlying symbolic understanding in ASD. Nevertheless, the potential relation between non-verbal IQ and symbolic word-picture-object mapping is highly intriguing, and should be explored by future research.

Due to the nature of this work – a small-scale pilot study – there are some significant limitations that should be noted. First, we caution against generalizing our conclusions across the autism spectrum because we focused on a specific group with limited language skills. More linguistically able children with ASD and Asperger syndrome may have an understanding of referential word-picture-object relations that is similar to TD peers. It is important to state that the children who participated in this study were representative of the population that this research is most relevant to: minimally verbal individuals with concomitant intellectual disabilities who receive and benefit from picture-based communication interventions. Moreover, the failure of children with ASD to generalize labels to differently colored category members cannot be attributed to insufficient language development, as all children had a receptive language age of at least 2 years (the age at which the shape bias emerges in TD children; Landau et al., 1988).

Second, the absence of a difference between the iPad and picture book mediums does not rule out the possibility that each affords different opportunities to support learning, which might be usefully exploited for children with ASD and other groups. We deliberately chose to minimize differences between the two formats in order to control for other variables that might influence performance. However, as noted above, we see the potential for the iPad to be used adaptively to provide varied repetition when required to maximize learning. In addition, for the reasons discussed previously, we believe that comparisons of engagement during learning and the different ways to support learning processes would provide useful information about how each medium can be exploited for this group. It will also be valuable to consider potential barriers to learning via iPads, for example, children with and without ASD may be so focused on the interactive aspects (e.g., such as pressing buttons) when they use the device independently that they fail to encode the learning material. This could be measured by manipulating the level of intervention provided by the experimenter to redirect attention when completing learning exercises, and would inform the process of learning. Finally, we note that children completed just two trials with an iPad/picture book and single/multiple exemplars respectively. Thus, this study should be regarded as the first *preliminary* evidence drawn from group data that children with ASD can learn novel symbol–referent concepts from iPads as effectively as traditional picture-based methods. However, we advocate that future studies re-address the influences of medium and content using fully between-subjects designs and perhaps more flexible tasks that promote categorization (e.g., a sorting task rather than a traditional forced-choice task; see Hartley and Allen, 2014b).

In sum, we have shown that children with ASD can learn novel symbol–referent mappings with an iPad and that their learning is comparable to a traditional picture book. There is considerable variability within the ASD population and we advocate that future research exploits the flexible and individualized learning that the iPad offers to determine how technology can be exploited to foster improved language and communication outcomes for this population. Future research also needs to consider how the different social-communicative context of learning with an iPad vs. traditional media might influence other aspects of development.

## Conflict of Interest Statement

The authors declare that the research was conducted in the absence of any commercial or financial relationships that could be construed as a potential conflict of interest.

## Footnotes

1 Two children received raw scores that fell below the range with a standardized age equivalent (2.3 years). Thus, we conservatively assigned a receptive language score of exactly 2 years to these participants.

2 When necessary, the experimenter intervened to prevent children repeatedly pressing the buttons without attending to the stimuli by redirecting attention and restarting the trial(s) during which children were inattentive.
